# Nitric Oxide Is Required for Melatonin-Enhanced Tolerance against Salinity Stress in Rapeseed (*Brassica napus* L.) Seedlings

**DOI:** 10.3390/ijms19071912

**Published:** 2018-06-29

**Authors:** Gan Zhao, Yingying Zhao, Xiuli Yu, Felix Kiprotich, Han Han, Rongzhan Guan, Ren Wang, Wenbiao Shen

**Affiliations:** 1 College of Life Sciences, Laboratory Center of Life Sciences, Nanjing Agricultural University, Nanjing 210095, China; 2015116111@njau.edu.cn (G.Z.); 2017116114@njau.edu.cn (Y.Z.); 2016116109@njau.edu.cn (X.Y.); kiprotichfelix@yahoo.com (F.K.); martinhan956@gmail.com (H.H.); 2 National Key Laboratory of Crop Genetics and Germplasm Enhancement, Jiangsu Collaborative Innovation Center for Modern Crop Production, Nanjing Agricultural University, Nanjing 210095, China; guanrzh@njau.edu.cn; 3 Institute of Botany, Jiangsu Province and Chinese Academy of Sciences, Nanjing 210014, China; wangren@126.com

**Keywords:** Arabidopsis, *Brassica napus*, ion homeostasis, melatonin, NaCl stress, nitric oxide, redox homeostasis

## Abstract

Although melatonin (*N*-acetyl-5-methoxytryptamine) could alleviate salinity stress in plants, the downstream signaling pathway is still not fully characterized. Here, we report that endogenous melatonin and thereafter nitric oxide (NO) accumulation was successively increased in NaCl-stressed rapeseed (*Brassica napus* L.) seedling roots. Application of melatonin and NO-releasing compound not only counteracted NaCl-induced seedling growth inhibition, but also reestablished redox and ion homeostasis, the latter of which are confirmed by the alleviation of reactive oxygen species overproduction, the decreases in thiobarbituric acid reactive substances production, and Na^+^/K^+^ ratio. Consistently, the related antioxidant defense genes, *sodium hydrogen exchanger* (*NHX1*), and *salt overly sensitive 2* (*SOS2*) transcripts are modulated. The involvement *S*-nitrosylation, a redox-based posttranslational modification triggered by NO, is suggested. Further results show that in response to NaCl stress, the increased NO levels are strengthened by the addition of melatonin in seedling roots. Above responses are abolished by the removal of NO by NO scavenger. We further discover that the removal of NO does not alter endogenous melatonin content in roots supplemented with NaCl alone or together with melatonin, thus excluding the possibility of NO-triggered melatonin production. Genetic evidence reveals that, compared with wild-type Arabidopsis, the hypersensitivity to NaCl in *nia1/2* and *noa1* mutants (exhibiting null nitrate reductase activity and indirectly reduced endogenous NO level, respectively) cannot be rescued by melatonin supplementation. The reestablishment of redox homeostasis and induction of *SOS* signaling are not observed. In summary, above pharmacological, molecular, and genetic data conclude that NO operates downstream of melatonin promoting salinity tolerance.

## 1. Introduction

Soil salinity is a major factor that significantly influences global agricultural production [1]. High salinity (mainly NaCl) provokes two primary effects on plants, including ionic and oxidative effects [1,2,3,4]. In general, high NaCl stress disturbs the ionic environment of plant cells, notably forming a higher Na^+^/K^+^ ratio [5]. Plants usually remove excessive Na^+^ by Na^+^/H^+^ antiporters, and genetic evidence revealed that overexpressing these antiporter genes can improve salt tolerance [6,7]. For example, *SOS* signaling is a well-known pathway responsible for initiating transport of Na^+^ out of the cells, or activating an unknown transporter, thus leading to the sequestration of Na^+^ in the vacuole [8,9,10]. Another type of Na^+^/H^+^ antiporter belongs to the Na^+^/H^+^ exchanger (NHX) family, and constitutive overexpression of *NHX* can increase Na accumulation in vacuoles, and thus, enhance salt tolerance [10]. Meanwhile, a large number of reactive oxygen species (ROS), such as superoxide anion, hydrogen peroxide, and hydroxy1 radicals, are induced under salinity conditions [11]. To combat salt-induced oxidative stress, the enzymatic antioxidant system provides a highly efficient and specific ROS scavenging approach for plants. For example, superoxide dismutase (SOD), catalase (CAT), and guaiacol peroxidase (POD), are very important parts of this enzymatic system, and normally, plants decrease ROS by upregulating activities of these enzymes [11,12,13].

Rapeseed (*Brassica napus* L.) is one of the most widely cultivated oil crops in the world because of the healthy fatty acid composition of its oil and high protein content of its meal. It is classified as a moderate salinity-tolerant crop [14]. During the growth period, rapeseed plants are challenged by salt stress, and ionic and redox imbalance are two major effects associated with salinity toxicity [11,14,15,16]. As more land becomes salinized, the studying of related mechanisms (the reestablishment of ionic and redox balance) and the application of effective methods (including reclamation of saline soils by use of chemicals or plant growth-promoting bacteria, or by growing salt tolerant cultivars in the saline soils, etc.) for improving salt tolerance in rapeseed plants [14,15,16,17,18], are becoming increasingly significant [19,20,21,22,23,24,25].

Melatonin (*N*-acetyl-5-methoxytryptamine) was discovered, and isolated from the bovine pineal gland in 1958 [26]. With a large set of functions in animals (circadian rhythms, seasonal rhythms, and alleviating oxidative stress; [27,28,29,30]), this compound was also detected in plants, and used as both a plant growth regulator and a biostimulator to alleviate abiotic and biotic stresses, including salinity, cold, drought, chemical pollutants, and defense against bacterial pathogen infection [31,32,33]. Previous results revealed that exogenous application of melatonin not only increased endogenous melatonin levels, but also improved the salt tolerance in Arabidopsis, soybean, Chinese crab apple, rice, cucumber, and bermudagrass [19,20,34,35,36,37]. Above beneficial roles of melatonin are normally associated with enhanced activities of antioxidant enzymes, as well as upregulating transcripts of ion channel genes, or sugar and glycolysis metabolism-related genes [36,37]. However, the corresponding detailed mechanism, especially the crosstalk with other signaling components and related transduction cascade, is still not fully characterized.

It is well known that nitric oxide (NO), one of the important gasotransmitters controlling a diverse range of physiological functions in plants [38,39,40], can also enhance salinity tolerance [21,41,42,43,44]. Previous reports revealed that there are at least two major enzymatic sources of NO: a nitrate/nitrite-dependent pathway and an l-Arg-dependent pathway [38,39]. Further experiments with Arabidopsis single and triple mutants, exhibiting null nitrate reductase (NR) activity (*nia1/2*) and indirectly reduced endogenous NO level (*nitric oxide associated1*; *noa1*), revealed that NO production is associated with salinity tolerance in Arabidopsis [42,44,45]. Importantly, protein post-translational modification by *S*-nitrosylation was preliminarily used to explain the physiological functions of NO in both animals and plants [46], including adaptation against biotic and abiotic stresses in plants [41,47,48]. However, it is not clear whether NO-dependent *S*-nitrosylation is also associated with melatonin responses in plants.

Although previous pharmacological data showed the interplay between melatonin and NO leading to plant tolerance against NaCl stress in sunflower seedlings [49,50], no genetic study has yet provided definitive proof of a role of endogenous NO in melatonin signaling governing salinity tolerance. In this study, we firstly evaluated the role of NO in melatonin-triggered salinity tolerance in rapeseed seedlings by using pharmacological and biochemical approaches. The reestablishment of redox and ion homeostasis was confirmed. The involvement of *S*-nitrosylation is also discovered, suggesting that NO is involved in melatonin signaling as a downstream messenger. Afterwards, both *nia1/2* and *noa1* Arabidopsis mutants were utilized to investigate the relationship between NO and melatonin in salinity tolerance. We thus concluded that NO acts downstream of melatonin signaling to enhance tolerance against salinity.

## 2. Results

### 2.1. Salt Stress Stimulates Melatonin and NO Production

**Figure 1:**
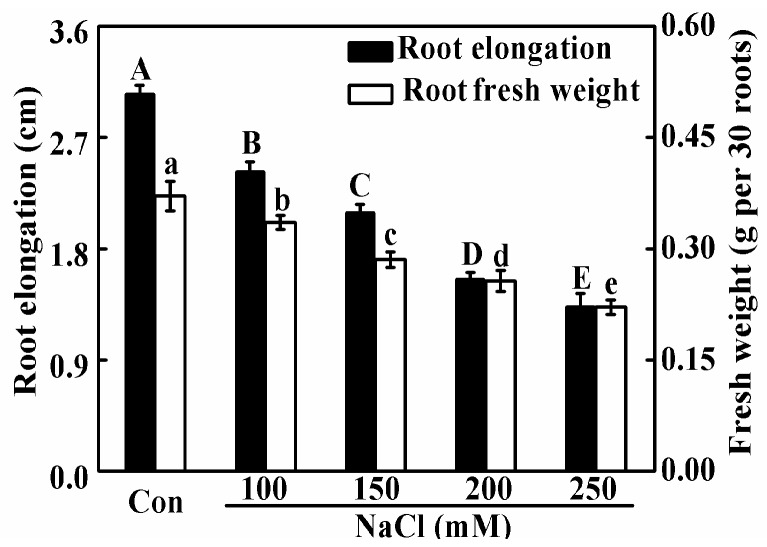
Growth inhibition of seedling roots upon NaCl stress. Three-day-old rapeseed seedlings were transferred to 100, 150, 200, and 250 mM NaCl for 2 days. Afterwards, the root elongation (left) and root fresh weight (right) were measured. The sample without chemicals was the control (Con). Values are means ± standard error (SE) of three independent experiments with at least three replicates for each. Bars with different letters are significant different at *p* < 0.05 according to Duncan’s multiple range test.

To assess the sensitivity of rapeseed seedling growth to NaCl stress, the effects of varying concentrations (100, 150, 200, and 250 mM) of NaCl on root growth were investigated. As shown in Figure 1, the exposure of seedlings to NaCl resulted in dose-dependent decreases in the root elongation and root fresh weight. Since approximate 50% inhibition in above parameters was observed in 200 mM NaCl-treated seedlings, this concentration of NaCl was applied in the following experiments.

**Figure 2:**
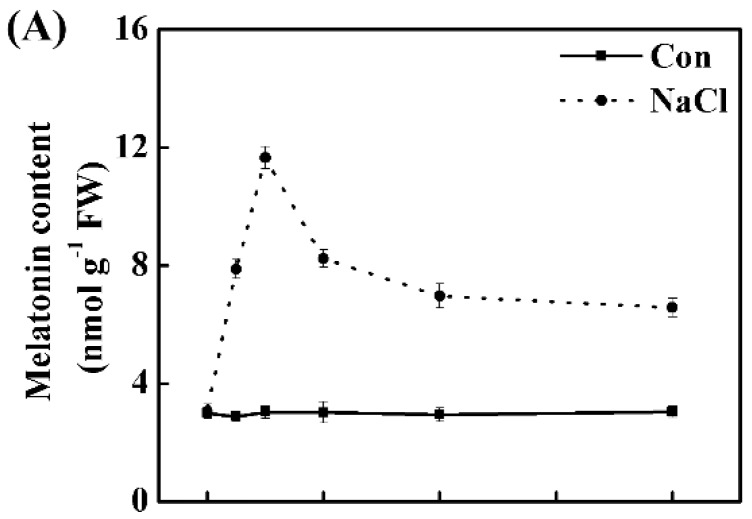

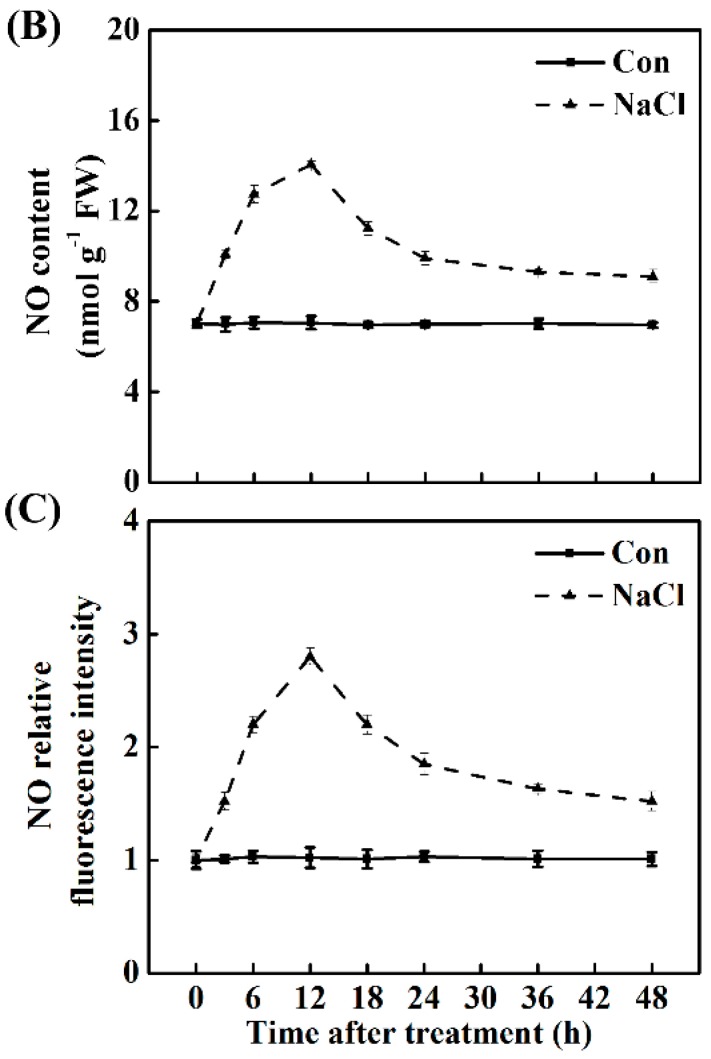
Changes in endogenous melatonin and nitric oxide (NO) levels in response to NaCl stress. Three-day-old rapeseed seedlings were transferred to 200 mM NaCl for 2 days. Meanwhile, melatonin (**A**) detected by enzyme-linked immunosorbent assay; and NO contents (**B**) determined by visible spectrophotography, and (**C**) determined by laser confocal scanning microscopy, and expressed as relative fluorescence intensity) in seedling roots were analyzed. The sample without chemicals was the control (Con). Values are means ± SE of three independent experiments with at least three replicates for each.

Griess reagent (visible spectrophotography) and laser scanning confocal microscopy (LSCM) with the specific probe (4-amino-5-methylamino-2′,7′-difluorofluorescein diacetate; DAF-FM DA), are the most frequently used methods for the determination of NO production in plants. Subsequently, the time course experiments for 48 h revealed the rapid burst of endogenous melatonin (Figure 2A; detected by enzyme-linked immunosorbent assay) and NO (Figure 2B,C; respectively determined by visible spectrophotography and LSCM) accumulation in NaCl-treated root tissues, peaking at 6 h and 12 h of stress, compared to the control sample (Con). We also noticed that the increases of melatonin and NO were still evident until 48 h, although both of them were decreased after the peaking points.

### 2.2. Melatonin and NO Alleviate NaCl-Induced Seedling Growth Inhibition

**Figure 3:**
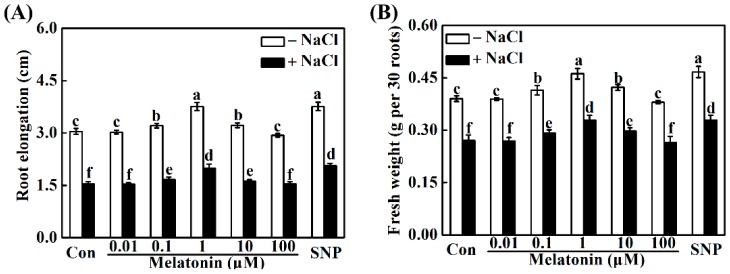
NaCl stress-triggered growth inhibition of seedling roots was alleviated by exogenous melatonin and sodium nitroprusside (SNP; a NO-releasing compound). Three-day-old rapeseed seedlings were pretreated with the indicated concentrations of melatonin or 10 μM SNP for 12 h, and then transferred to 200 mM NaCl for another 2 days. Afterwards, the root elongation (**A**) and root fresh weight (**B**) were measured. The sample without chemicals was the control (Con). Values are means ± SE of three independent experiments, with at least three replicates for each. Bars with different letters are significant different at *p* < 0.05 according to Duncan’s multiple range test.

It was well known that to discern the role of melatonin in the alleviation of salt stress, a dose–response study of exogenous melatonin in vitro was firstly established. As shown in Figure 3, we observed that the addition of melatonin (0.1, 1, and 10 μM) not only promoted seedling root growth under the normal growth condition, but also differentially alleviated the growth inhibition in roots triggered by NaCl stress, while no significant rescuing effects were observed in 0.01 and 100 μM melatonin-pretreated seedlings. Among these pretreatments, the responses of 1 μM melatonin was maximal, and this concentration was further applied in the following test.

Meanwhile, the treatment with three types of NO-releasing compounds, namely sodium nitroprusside (SNP), diethylamine NONOate (NONOate), and *S*-nitrosoglutathione (GSNO), produced similar positive responses in the stressed condition (Figure 3 and Supplementary Materials Figure S1). While, old SNP (a negative control of SNP) failed to influence root growth inhibition. Above results thus suggested the beneficial role of exogenous NO in the plant tolerance against salinity stress. Considering the cost of chemicals, SNP was used as a NO-releasing compound in the following experiment.

### 2.3. PTIO-Dependent Removal of NO Production Impairs the Response of Melatonin

**Figure 4:**
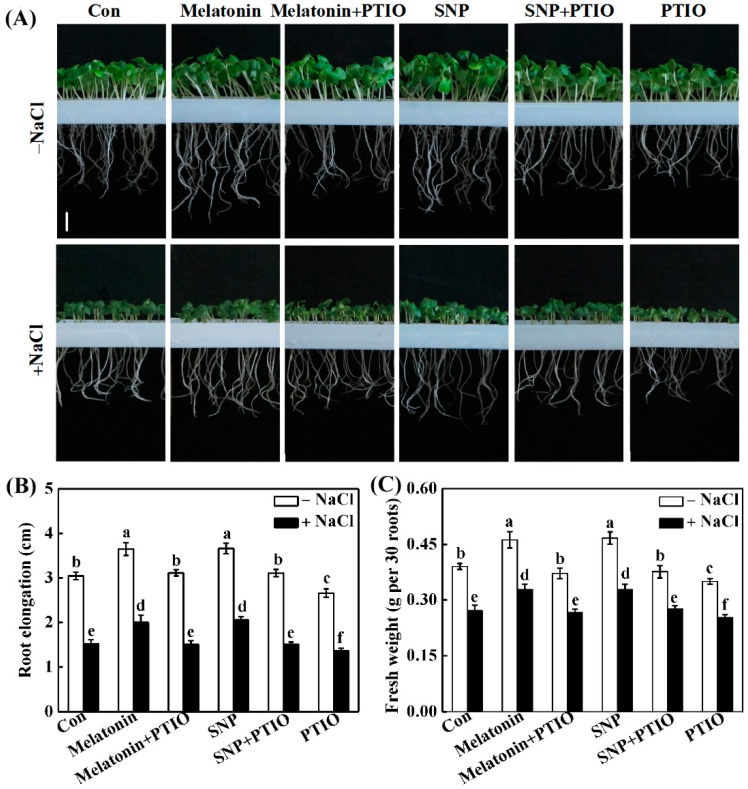
Exogenous melatonin-alleviated root growth inhibition caused by NaCl stress was sensitive to the removal of NO. Three-day-old rapeseed seedlings were pretreated with 1 μM melatonin, 10 μM SNP, 200 μM PTIO, alone or their combinations for 12 h, and then transferred to 200 mM NaCl for 2 days. Afterwards, corresponding photographs were taken ((**A**); bar: 1 cm). The root elongation (**B**) and root fresh weight (**C**) were measured. The sample without chemicals was the control (Con). Values are means ± SE of three independent experiments with at least three replicates for each. Bars with different letters are significant different at *p* < 0.05 according to Duncan’s multiple range test.

To assess the possible link between melatonin and NO in the alleviation of NaCl stress, the effects of the NO scavenger 2-phenyl-4,4,5,5-tetramethylimidazoline-1-oxyl-3-oxide (PTIO), on the abovementioned melatonin and SNP responses, were investigated and compared. The results shown in Figure 4 revealed that both melatonin- and SNP-alleviated root growth inhibition was greatly reduced in the presence of PTIO, which was similar to the phenotypes in NaCl-stressed alone conditions. In comparison with NaCl stress, the addition of PTIO aggravated root growth inhibition.

**Figure 5:**
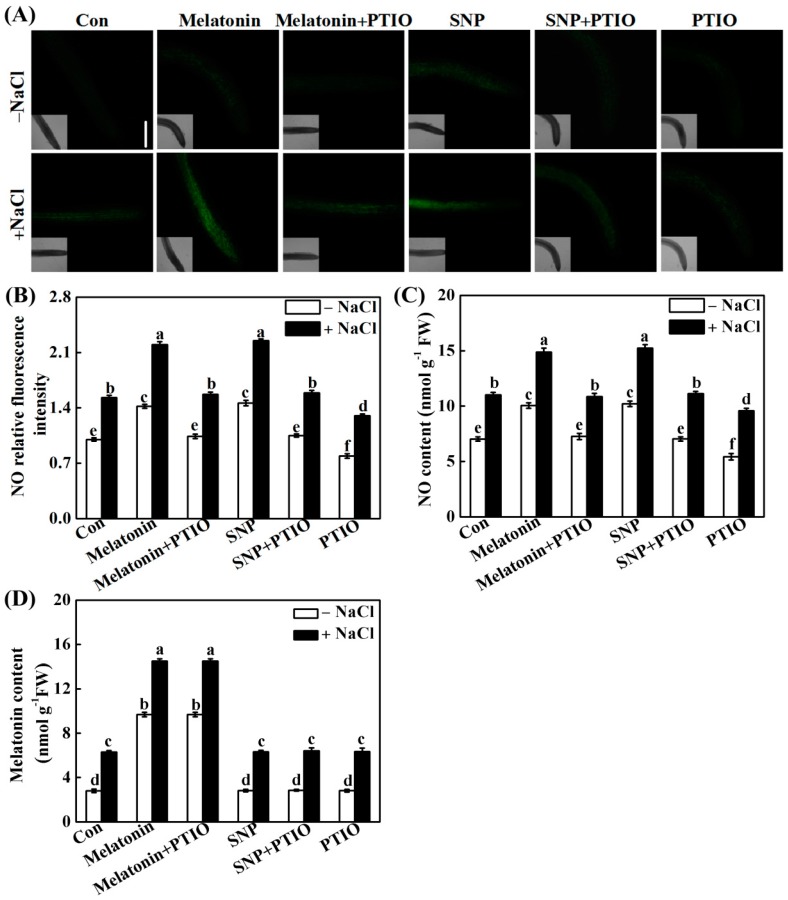
The removal of NO did not alter endogenous melatonin level, but melatonin triggered NO production. Three-day-old rapeseed seedlings were pretreated with 1 μM melatonin, 10 μM SNP, 200 μM PTIO, alone or their combinations for 12 h, and then transferred to 200 mM NaCl for another 2 days. Afterwards, NO ((**A**); determined by laser confocal scanning microscopy; (**C**); determined by visible spectrophotography) and melatonin contents ((**D**); detected by enzyme-linked immunosorbent assay) in root tissues were detected. Scale bar = 1 mm. DAF-FM DA fluorescence densities according to (**A**) were also given (**B**). The sample without chemicals was the control (Con). Values are means ± SE of three independent experiments with at least three replicates for each. Bars with different letters are significant different at *p* < 0.05 according to Duncan’s multiple range test.

The role of NO in melatonin-enhanced salinity tolerance was further examined by monitoring NO synthesis in response to applied melatonin and SNP in the presence or absence of PTIO. Similar to the response of SNP, a significant increase in NO-induced fluorescence was observed in stressed seedling roots compared with the control tissue, demonstrating melatonin-mediated NO production (Figure 5A,B). Importantly, melatonin-induced NO synthesis was abolished by co-incubation with PTIO, correlating these data with those from phenotypic analysis (Figure 4). The above results were further confirmed by Griess reagent method (Figure 5C). Together, the pharmacological evidence revealed that PTIO-dependent removal of NO production impairs the response of melatonin.

### 2.4. NO Does Not Alter Melatonin Synthesis

To further confirm above hypothesis, the effects of SNP and PTIO on endogenous melatonin levels were analyzed. Unlike the inducible responses of exogenous melatonin, the treatment with SNP had no effect on either basal or NaCl-induced melatonin production (Figure 5D). Interestingly, the co-incubation with PTIO did not influence melatonin levels in response to either melatonin or SNP when applied exogenously, no matter if seedlings were with or without the treatment of NaCl.

### 2.5. Redox Balance Is Reestablished by Melatonin via NO

**Figure 6:**
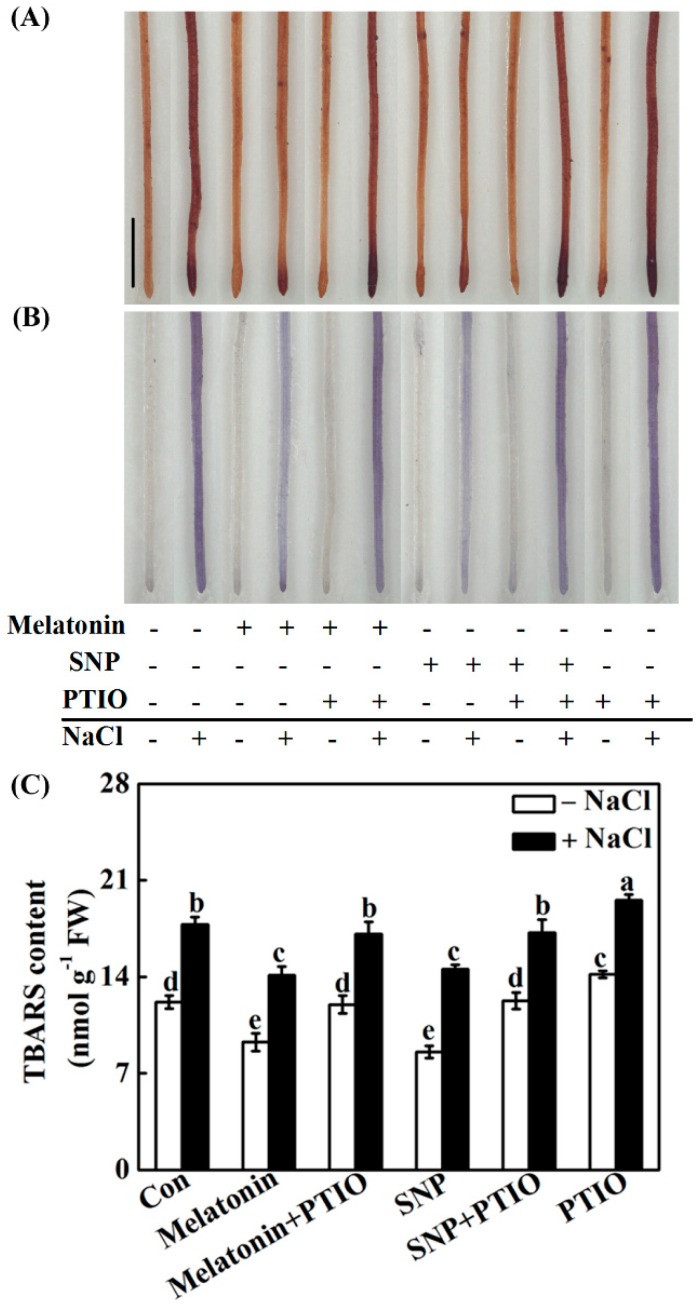
Redox balance was reestablished by melatonin via NO. Three-day-old rapeseed seedlings were pretreated with 1 μM melatonin, 10 μM SNP, 200 μM PTIO, alone or their combinations for 12 h, and then transferred to 200 mM NaCl for another 2 days. Afterwards, seedling roots were stained with DAB (**A**) and NBT (**B**) to detect H_2_O_2_ and O_2_^−^. Scale bar = 1 cm. TBARS content (**C**) were also determined. The sample without chemicals was the control (Con). Values are means ± SE of three independent experiments with at least three replicates for each. Bars with different letters are significant different at *p* < 0.05 according to Duncan’s multiple range test.

To unravel the molecular mechanism underlying melatonin-triggered salinity tolerance, subsequent histochemical detection of hydrogen peroxide (H_2_O_2_; DAB staining) and superoxide anion (O_2_^–^; NBT staining) was applied. Similar to the positive responses of SNP, NaCl-induced H_2_O_2_ and O_2_^–^ overproduction in roots, confirmed by the dark brown (Figure 6A) and purple-blue (Figure 6B) color precipitates, was differentially abolished by melatonin. Contrasting results were observed when PTIO was added together. These were in accordance with the results of TBARS contents (Figure 6C).

**Figure 7:**
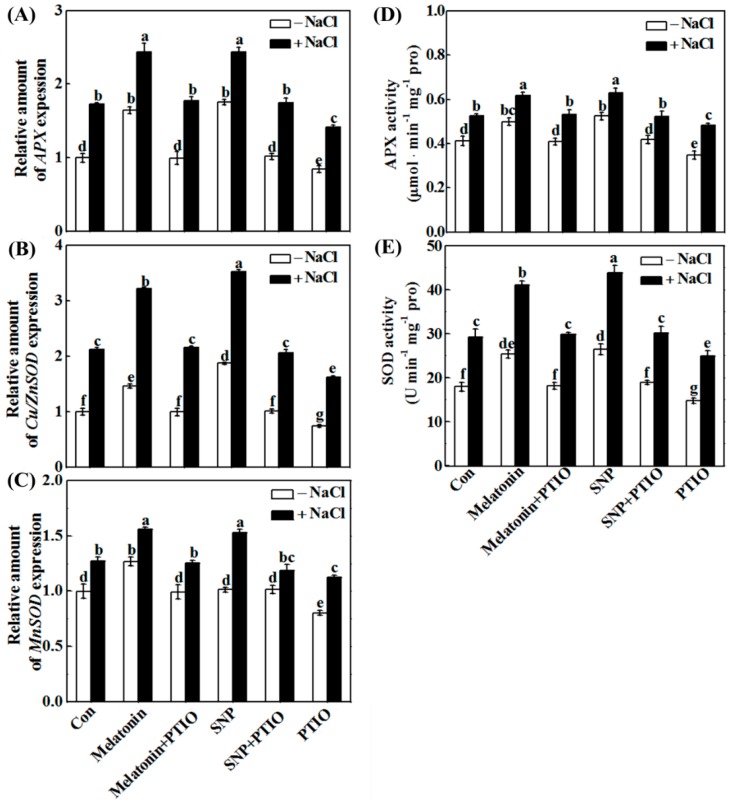
Antioxidant genes and corresponding enzymatic activities were modulated by melatonin-mediated NO. Three-day-old rapeseed seedlings were pretreated with 1 μM melatonin, 10 μM SNP, 200 μM PTIO, alone or their combinations for 12 h, and then transferred to 200 mM NaCl for another 12 h (**A**–**C**) or 2 days (**D**,**E**). Then, the mRNA expression of *APX* (**A**), *Cu/ZnSOD* (**B**), and *MnSOD* (**C**) in root tissues was analyzed by qPCR. The activities of ascorbate peroxidase (APX; (**D**)) and superoxide dismutase (SOD; (**E**)) were determined. The sample without chemicals was the control (Con). Values are means ± SE of three independent experiments with at least three replicates for each. Bars with different letters are significant different at *p* < 0.05 according to Duncan’s multiple range test.

Molecular and biochemical experiments revealed that treatment with PTIO almost completely blocked the increases in the expression of the antioxidant genes *APX*, *MnSOD*, *Cu/ZnSOD* (Figure 7A–C), and the activities of APX and SOD (Figure 7D,E) in NaCl-stressed root tissues. Combined with the results in histochemical detection and TBARS content analysis (Figure 6), these clearly suggested the requirement of NO in melatonin-reestablished redox balance.

### 2.6. Melatonin Modulates Ion Homeostasis via NO

**Figure 8:**
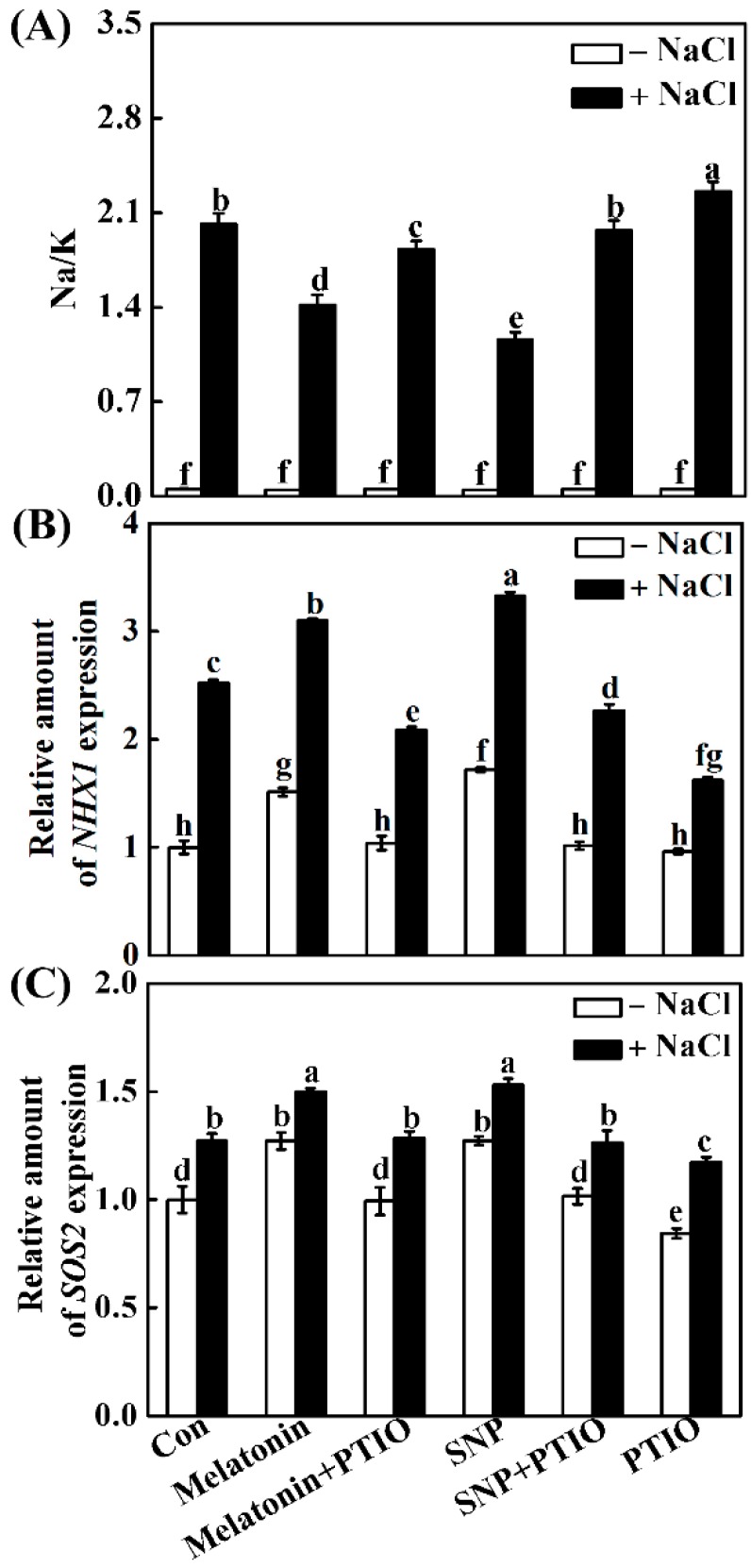
Melatonin modulated ion homeostasis via NO. Three-day-old rapeseed seedlings were pretreated with 1 μM melatonin, 10 μM SNP, 200 μM PTIO, alone or their combinations for 12 h, and then transferred to 200 mM NaCl for another 2 days (**A**) or 12 h (**B**,**C**). Afterwards, Na^+^ to K^+^ ratio (**A**) in seedling roots were detected by ICP-OES. The mRNA expression of *NHX1* (**B**) and *SOS2* (**C**) were analyzed by qPCR. The sample without chemicals was the control (Con). Values are means ± SE of three independent experiments with at least three replicates for each. Bars with different letters are significant different at *p* < 0.05 according to Duncan’s multiple range test.

The keeping of ion homeostasis is essential for plants to resist salt stress. Therefore, the effects of melatonin and NO, as well as their interplay on ion homeostasis, were investigated. Compared with the control, NaCl stress significantly increased the Na^+^ accumulation and decreased the K^+^ accumulation, thus leading to a higher Na^+^/K^+^ ratio (Figure 8A) in seedling roots. By contrast, the addition of melatonin and SNP helped seedling roots to reduce the accumulation of Na^+^ and improve K^+^ assimilation, resulting in lower Na^+^/K^+^ ratio, compared with NaCl stressed alone, both of which could be reversed by PTIO.

To confirm the cause of this phenomenon, transcripts of Na^+^ transporter *NHX1* and *SOS2* were analyzed. As expected, changes in *NHX1* and *SOS2* were consistent with the results in Na^+^/K^+^ ratio. For example, the removal of endogenous NO by PTIO completely impaired the effects of melatonin and SNP on activating *NHX1* and *SOS2* mRNA (Figure 8B,C).

### 2.7. The Possible Involvement of NO-Dependent S-Nitrosylation

**Figure 9:**
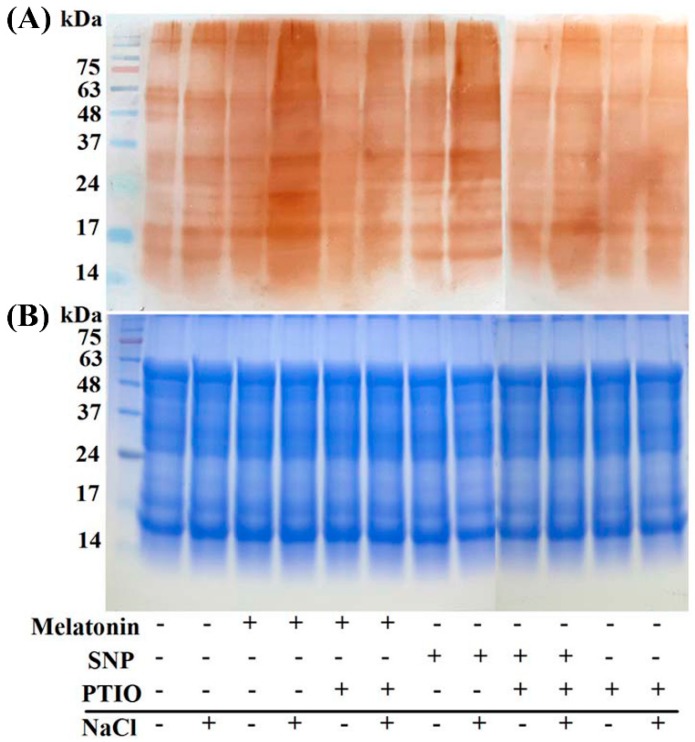
Immunoblot analysis of the total *S*-nitrosylated protein. Three-day-old rapeseed seedlings were pretreated with 1 μM melatonin, 10 μM SNP, 200 μM PTIO alone, or in various combinations, for 12 h, and then transferred to 200 mM NaCl for another 2 days. The sample without chemicals was the control (Con). Afterwards, proteins were extracted from seedling roots, and subjected to the modified biotin switch method. The labelled proteins were detected using protein blot analysis with antibodies against biotin (**A**). Numbers on the left of the panels indicate the position of the protein markers in kDa. A Coomassie Brilliant Blue-stained gel (**B**) is present to show that equal amounts of proteins were loaded.

An important bioactivity of NO is implemented by regulating the activity of targeted proteins through *S*-nitrosylation, a redox-based posttranslational modification. For further confirming the involvement of NO in above melatonin responses, the profiles in *S*-nitrosylation were analyzed by using the modified biotin switch technique. Figure 9 showed that both melatonin and SNP increased *S*-nitrosylation under NaCl stress, which could be partially blocked by the PTIO-dependent removal of NO production. Alone, PTIO supplementation slightly decreased *S*-nitrosylation, compared to the control samples.

### 2.8. Genetic Evidence Reveals That NO Is Required for Melatonin-Induced Salinity Tolerance

**Figure 10:**
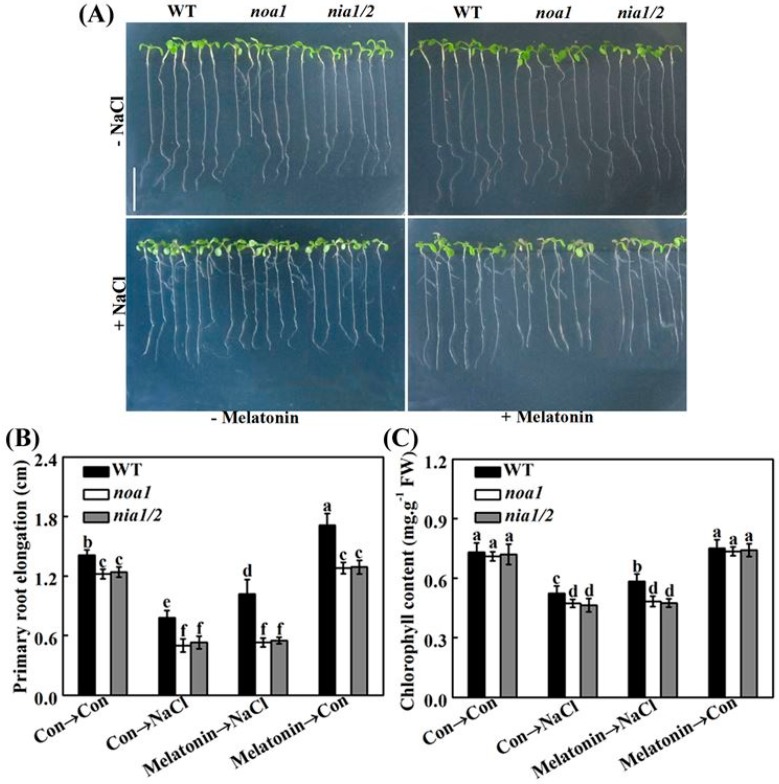
Genetic evidence supported the requirement of NO in melatonin-alleviated NaCl stress. Five-day-old wild-type (WT), and *noa1* and *nia1/2* mutant plants were grown on MS medium supplemented with 1.0 μM melatonin for 5 days, and then transplanted to medium in the presence or absence of 125 mM NaCl for another 5 days. Primary root elongation (**B**) and total chlorophyll content in leaves (**C**) were then determined to assess changes in salt tolerance described in (**A**). Control seedlings (Con) were grown in MS medium alone. Scale bar = 1 cm. Data are means ± SE of three independent experiments with at least three replicates for each. Bars with different letters are significant different at *p* < 0.05 according to Duncan’s multiple range test.

To complement above results, we evaluated the role of NO in melatonin-triggered salinity tolerance by using Arabidopsis wild-type (WT) and mutants exhibiting null nitrate reductase (NR) activity (*nia1/2*) and indirectly reduced endogenous NO level (*nitric oxide associated1*; *noa1*). Figure 10A shows WT and *nia1/2* and *noa1* mutants challenged by NaCl stress in the presence or absence of melatonin. As expected, the *nia1/2* and *noa1* mutants exhibited more sensitivity to salinity stress than WT, as measured by the responses in primary root elongation (Figure 10B) and chlorophyll content (Figure 10C) in seeding leaves. It was subsequently observed that the NaCl-induced toxicity in WT plants was obviously rescued by the pretreatment with melatonin. By contrast, no significant rescuing responses were observed in the stressed *nia1/2* and *noa1* mutants with melatonin. Since there are at least two distinct pathways responsible for NO synthesis in plants, the NR- and l-Arg-dependent pathways, our genetic evidence further confirmed the central role of NO in salt tolerance triggered by melatonin.

**Figure 11:**
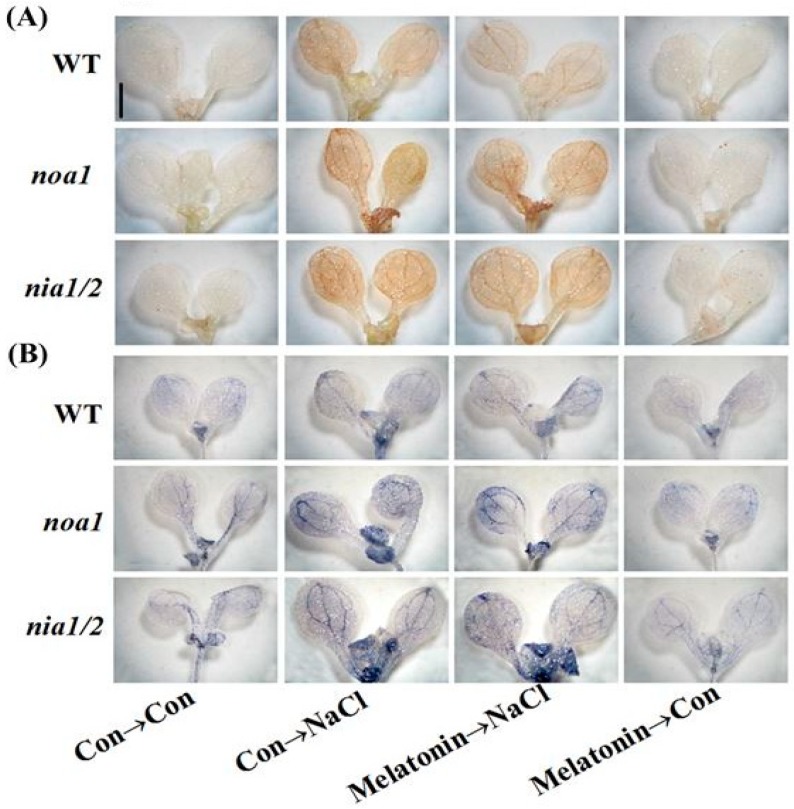
Genetic evidence revealed that redox balance was reestablished by melatonin via NO. Five-day-old wild-type (WT), *noa1*, and *nia1/2* mutant plants were grown on MS medium supplemented with 1.0 μM melatonin for 5 days, and then transplanted to medium in the presence or absence of 125 mM NaCl for another 5 days. Control seedlings (Con) were grown in MS medium alone. Afterwards, seedlings were stained with DAB (**A**) and NBT (**B**) to detect H_2_O_2_ and O_2_^−^. Scale bar = 1 mm.

**Figure 12:**
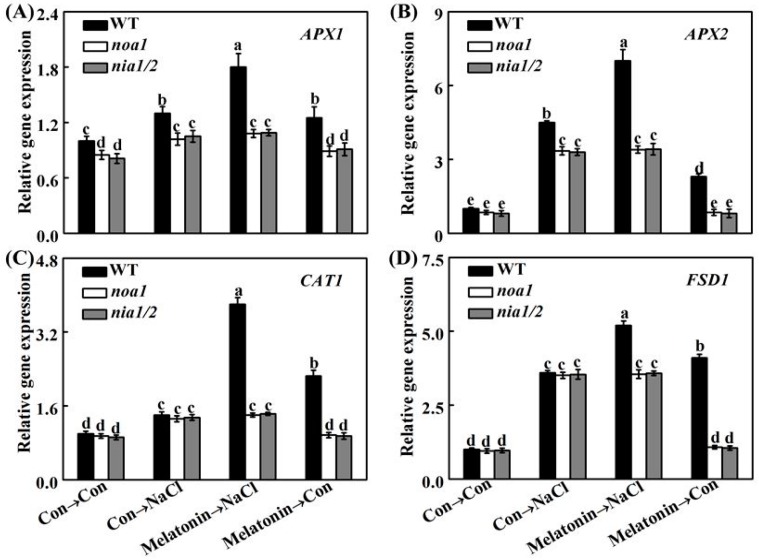
Changes in antioxidant gene expression. Five-day-old wild-type (WT), *noa1*, and *nia1/2* mutant plants were grown on MS medium supplemented with 1.0 μM melatonin for 5 days, and then transplanted to medium in the presence or absence of 125 mM NaCl for another 24 h. The mRNA expression of *APX1* (**A**), *APX2* (**B**), *CAT1* (**C**), and *FSD1* (**D**) in root tissues were analyzed by qPCR. Control seedlings (Con) were grown in MS medium alone. Data are means ± SE of three independent experiments with at least three replicates for each. Bars with different letters are significant different at *p* < 0.05 according to Duncan’s multiple range test.

**Figure 13:**
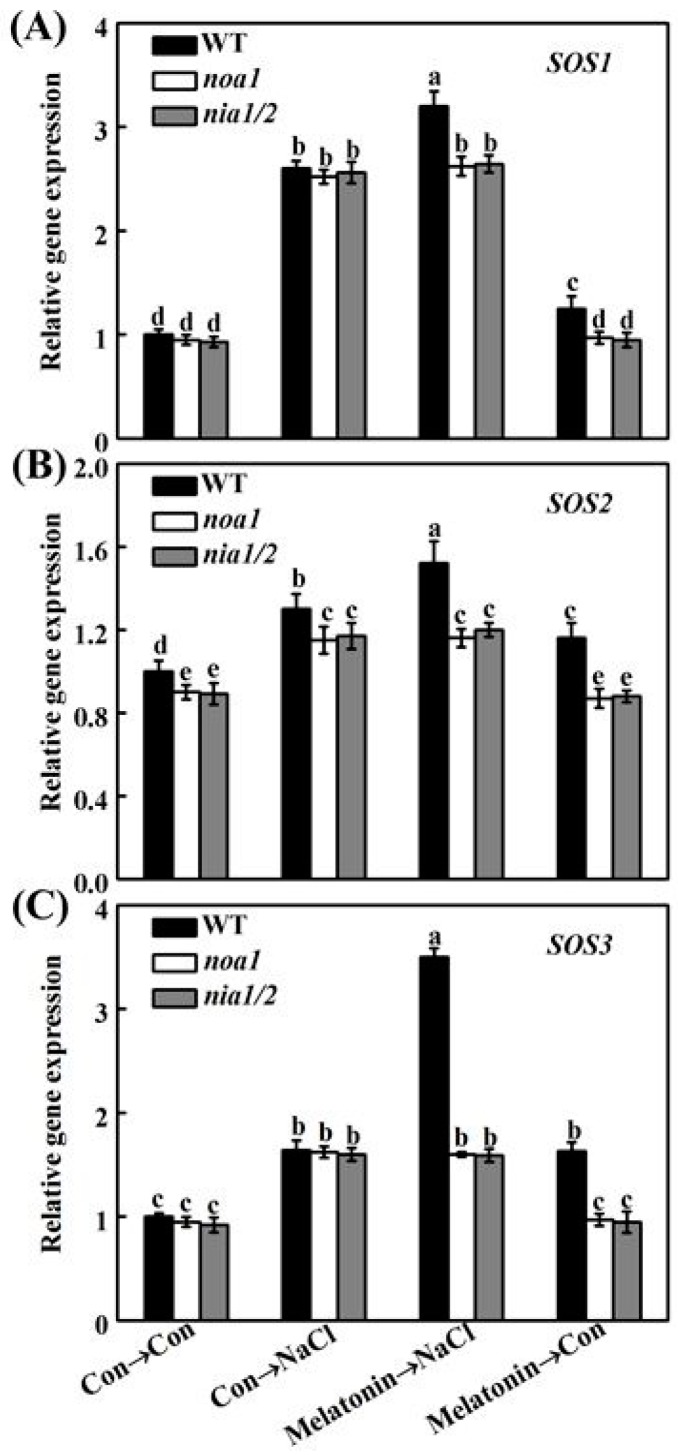
Gene evidence indicated that *SOS* signaling pathway was regulated by melatonin via NO. Five-day-old wild-type (WT), *noa1*, and *nia1/2* mutant plants were grown on MS medium supplemented with 1.0 μM melatonin for 5 days, and then transplanted in medium in the presence or absence of 125 mM NaCl for another 24 h. The mRNA expression of *SOS1* (**A**), *SOS2* (**B**), and *SOS3* (**C**) in root tissues were analyzed by qPCR. Control seedlings (Con) were grown in MS medium alone. Data are means ± SE of three independent experiments with at least three replicates for each. Bars with different letters are significant different at *p* < 0.05 according to Duncan’s multiple range test.

In order to further assess whether melatonin-reestablished redox and ion homeostasis is associated with NO signaling, histochemical detection and molecular approach were adopted. As shown in Figure 11, unlike the WT plants, the NaCl-triggered H_2_O_2_ and O_2_^–^ overproduction in *nia1/2* and *noa1* mutants was largely insensitive to melatonin supplementation. Consistently, NaCl-induced *APX1*, *APX2*, *CAT1*, and *FSD1* transcripts were strengthened by melatonin in wild-type (Figure 12). In comparison, no such induction conferred by melatonin appeared in the mutant plants upon NaCl stress. Molecular evidence further showed that *SOS1*, *SOS2*, and *SOS3* transcripts were upregulated in wild-type and mutant seedlings upon NaCl stress (Figure 13). In the presence of melatonin, above induction was more pronounced in wild-type, but not in *nia1/2* and *noa1* mutants. Combined with the changes in phenotypes (Figure 10), the above genetic evidence thus suggested that endogenous NO mainly produced by NR and NOA1 is required for melatonin-triggered salinity tolerance in Arabidopsis, and the reestablished redox and ion homeostasis were suggested.

## 3. Discussion

In this work, the molecular basis of melatonin-mediated plant salinity tolerance was investigated. The pharmacological and genetic data presented here show that rapeseed seedlings respond to salinity stress by increasing the concentration of melatonin and NO successively, and that NO is required for the melatonin-mediated plant tolerance against salinity stress.

First, the result shows that an increase in the concentration of melatonin followed by the induction of NO production, is one of the earliest responses involved in the signaling transduction elicited by NaCl stress in rapeseed seedlings (Figure 2). Although the major source(s) of NO have not been investigated in rapeseed seedlings, a cause-effect relationship between melatonin and NO production in salinity tolerance was further established.

Compared to the beneficial role of melatonin in the terms of the alleviation of rapeseed root growth inhibition caused by NaCl stress (Figure 3 and Figure S1), the application of SNP, NONOate, and GSNO, three well-known NO-releasing compounds, could result in the similar responses. The above effect was not found in seedlings supplemented with old SNP, a negative control of SNP, which contains no NO, but nitrate, nitrite, and ferrocyanide [51,52]. These results confirmed the previous conclusion that NO acts as an important gaseous molecule with multiple biological functions in plants, especially in salt tolerance [34,35,36,37,38].

Previously, the interplay between melatonin and NO in plant responses against stress has been controversial [53]. For example, pharmacological data showed that exogenous melatonin induces the production of NO in alkaline stressed tomato seedlings, and NO might be a downstream signal involved in the enhancement of tomato tolerance against alkaline stress triggered by melatonin [54]. A similar conclusion was obtained in Arabidopsis innate immunity against bacterial pathogen (Pst DC3000) [33]. In the present study, mimicking the action of SNP, the increased NO synthesis in response to melatonin in NaCl-stressed rapeseed seedling roots is demonstrated (Figure 5A–C). The removal of endogenous NO by PTIO, however, impaired the effects of melatonin and SNP. Most importantly, the above processes were correlated to the phenotypic responses, showing that the enhancement of plant tolerance against NaCl stress by melatonin is associated with endogenous NO levels (Figure 4). The above results were consistent with reported by Wen et al. [55], in which they found that the increased NO levels caused by the inhibition of *S*-nitrosoglutathione reductase (GSNOR), which could negatively regulate the NO accumulation in plants [56], were involved in melatonin-triggered adventitious rooting in cucumber plants. Since abiotic stress could trigger adventitious root formation, an important phenotype of the stress-induced morphogenic response (SIMR) in plants [57], our results further confirmed the biological roles of NO in both development and plant responses against stress [38]. Meanwhile, the removal of endogenous NO by PTIO does not alter endogenous melatonin synthesis in roots supplemented with melatonin or NaCl, neither alone or in different combinations (Figure 5D), thus excluding the possibility of NO-triggered melatonin production. By contrast, several previous reports have provided pharmacological evidence indicating that NO could stimulate endogenous melatonin accumulation in sunflower seedling cotyledons as a long-distance signaling response under salt stress [49,50]. NO-dependent melatonin synthesis in cadmium-stressed rice seedlings were also observed [58]. The above differences may reflect the complexity of melatonin signaling in plants [52], and the interplay between melatonin and NO might be dependent on the doses of stresses and exposure times, and even different plant species.

Lozano-Juste and Leŏn [45] reported that *nia1/2* and *noa1* mutants were more sensitive to NaCl-inhibited germination than wild-type seeds. Thus, we obtained these mutants to further study the role of endogenous NO in Arabidopsis salinity tolerance achieved by melatonin. Figure 10 shows that *nia1/2* and *noa1* seedlings seemed to be more sensitive than wild-type to NaCl stress, in terms of primary root elongation and chlorophyll degradation (in particular). Most importantly, exogenous melatonin-enhanced salt tolerance was markedly impaired in above Arabidopsis mutants, which were impaired in NIA/NR- and AtNOA1-dependent NO biosynthesis [42,44,45,51,59]. Thus, this genetic evidence supported the requirement of NO in melatonin-alleviated NaCl stress, at least in our experimental conditions, although the possibility of the direct scavenging or the inhibition of NO synthesis by melatonin could not be easily ruled out [60,61,62].

Upon NaCl stress, redox imbalance caused by ROS overproduction normally occurred in plants, thus leading to growth stunt and even cell death [1,2,3,4]. Further genetic evidence strongly revealed that plant tolerance against NaCl stress is closely associated with a more efficient antioxidant defense [13,20,35,63]. Previously, the antioxidant properties of melatonin against the overproduction of ROS have been confirmed in plant responses against salinity stress [34,35,36,37]. Similarly to the above discoveries, our results revealed that melatonin counteracted NaCl stress-induced oxidative damage in rapeseed seedlings, which could be confirmed by the decreased ROS production and lipid peroxidation (Figure 6), as well as the induction of representative antioxidant gene expression, including *APX*, *Cu/ZnSOD*, and *MnSOD* (Figure 7A–C). By using biochemical determination, it was further suggested that melatonin was able to increase APX and SOD activities (Figure 7D,E), not only at the transcriptional levels. By contrast, the above changes were prevented by PTIO, an NO scavenger [41,42], indicating that NO is involved in the reestablishment redox balance triggered by melatonin in NaCl stressed rapeseed seedlings. Consistently, we further showed that melatonin was able to reestablish redox balance in Arabidopsis wild-type seedlings upon NaCl stress, but was ineffective in *nia1/2* and *noa1* plants, two NO-deficient mutants (Figure 11 and Figure 12), both of which were used to dissect physiological function of NO in plants [42,44,45]. Together, the above genetic evidence further confirmed that the counteracting effect of melatonin on oxidative damage induced by NaCl stress is NO-dependent.

Maintenance of ion homeostasis, in particular, the Na^+^ to K^+^ ratio, is another important approach for plants to resist NaCl stress [5,63]. Previous results showed that SOS1, a Na^+^/H^+^ antiporter, mainly mediated Na efflux, and the activation of SOS2/SOS3 complex can regulate *SOS1* gene expression [63,64]. The reestablishment of ion homeostasis by NO was previously confirmed in NaCl-stressed reed plants, showing that the NaCl-enhanced Na^+^ to K^+^ ratio is decreased by NO [41]. The application with melatonin exhibited a similar action [34,35], which was confirmed in rapeseed seedlings upon NaCl stress (Figure 8A). Interestingly, changes in rapeseed *NHX1* and *SOS2* transcripts were consistent with the results in Na^+^/K^+^ ratio (Figure 8B,C). However, the above changes were impaired by the removal of NO, suggesting the NHX-mediated Na^+^-sequestration [10] and SOS-mediated Na^+^ efflux [64] triggered by NO, might be two important strategies for plant tolerance against NaCl stress when supplemented with melatonin. The above conclusion was partially supported by the changes of *SOS1*, *SOS2*, and *SOS3* transcripts in wild-type, and *nia1/2* and *noa1* Arabidopsis mutants (Figure 13).

NO-mediated *S*-nitrosylation, a redox-related modification of cysteine thiol, is regarded as one of the important post-translational modifications to regulate enzyme activity and interactions among proteins in animals [46]. In higher plants, the formation of *S*-nitrosylation is associated with a wide range of physiological responses, including stress tolerance and root organogenesis [40,47,48,62]. Although a previous paper points to the possible involvement of NO-dependent *S*-nitrosylation in melatonin signaling [33], no study has yet provided definitive proof. In the subsequent work, we discovered that NaCl-induced *S*-nitrosylation was intensified by melatonin and SNP, but impaired by the removal of NO, respectively (Figure 9). Although we have not investigated the detailed target proteins of *S*-nitrosylation, our results strengthened the role of NO in melatonin responses. 

**Figure 14:**
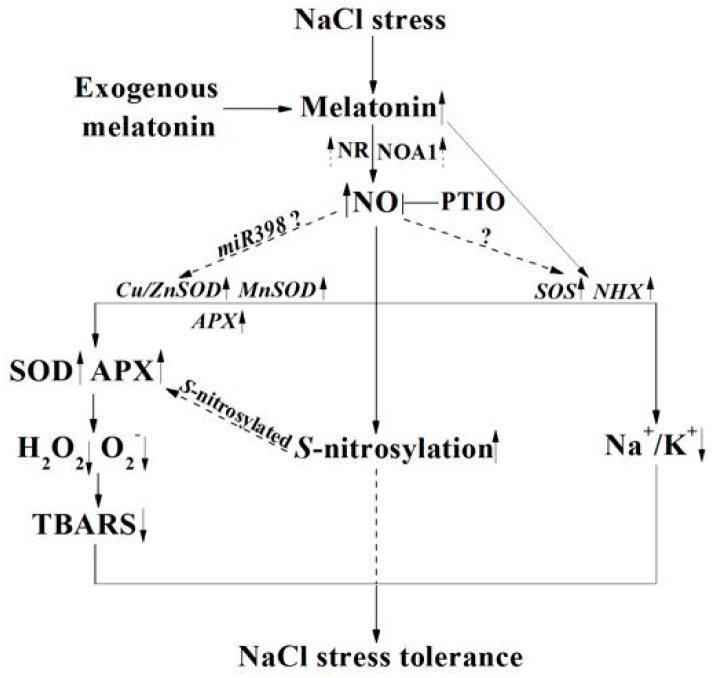
A model depicting the requirement of NR- and NOA1-dependent NO in melatonin-enhanced tolerance against salinity. The reestablishment of redox and ion homeostasis was involved. Dashed lines denote indirect or still undescribed pathways, including *miRNA398*-modulated gene expression [65] and *S*-nitrosylated antioxidant enzymes (APX, [48]).

Overall, we here presented the first genetic and pharmacological evidence showing the involvement of NO in melatonin signaling in salinity tolerance, and the model was shown in Figure 14. This model proposes that, upon NaCl stress, the increased melatonin triggers a signaling cascade that leads to an induction in NR- and NOA1-dependent NO concentration, thus resulting in the enhancement of salt stress tolerance. Meanwhile, the reestablishment of redox and *SOS*-mediated ion homeostasis are regarded as the important mechanism. NO-dependent *S*-nitrosylation was also illustrated in melatonin responses, and this is a new finding. Thus, the possibility that *miRNA398* modulated antioxidant gene expression [65] and *S*-nitrosylation targeted APX [48], in the above melatonin-mediated NO action, could not be easily ruled out. Further investigation may reveal melatonin-mediated NO-targeted proteins for genetic modification following biotechnological approaches, ultimately aiming to enhance plant tolerance against NaCl stress. Additionally, related studies using potted or field grown rapeseed plants incubated in soil/potting media with a prolonged experimental time would further help in understanding the melatonin and NO application in improving abiotic stress tolerance, thus providing potential benefits in agriculture.

## 4. Materials and Methods

### 4.1. Chemicals

All chemicals were purchased from Sigma-Aldrich (St. Louis, MO, USA) unless stated otherwise. The chemicals used for treatment were melatonin, sodium nitroprusside (SNP, a NO-releasing compound), 2-phenyl-4,4,5,5,-tetramethylimidazoline-1-oxyl-3-oxide (PTIO, a scavenger of NO), diethylamine NONOate (NONOate, a NO donor), and *S*-nitrosoglutathione (GSNO, a NO donor). An SNP solution was used as a negative control of SNP, by maintaining a separated solution of SNP for at least 10 days in light, in a specific open tube, to eliminate all NO [66]. The concentrations of chemicals used in this study were determined in pilot experiments from which maximal induced responses were obtained.

### 4.2. Plant Materials, Growth Condition, and Experimental Design

Rapeseed (*Brassica napus* L. zhongshuang 11) were kindly supplied by Chinese Academy of Agricultural Sciences. Rapeseed were surface-sterilized with 5% NaClO for 10 min, and rinsed comprehensively in distilled water, then germinated for 2 days at 25 °C in the darkness. Subsequently, the uniform seedlings were cultured with half-strength Hoagland solution in an illuminating incubator (16 h light with a light intensity of 200 μmol·m^−2^·s^−1^, 25 ± 1 °C, and 8 h dark, 23 ± 1 °C). Three-day-old rapeseed seedlings were treated with the indicated chemicals as shown in legends. Three independent experiments with at least three replicates for each were performed, and 30 samples were included in each replicate. Afterwards, rapeseed plants were photographed, and seedling roots were measured immediately after various treatments, or sampled for other analysis.

*Arabidopsis thaliana noa1* (CS6511, Col-0) and *nia1/2* (CS2356, Col-0) mutants were obtained from the Arabidopsis Biological Resource Center (http://www.arabidopsis.org/, 5 June 2018). Seeds were surface-sterilized by sodium hypochlorite and rinsed three times with sterile water, then cultured on the solid Murashige and Skoog (MS, pH 5.8) medium containing 1% (*w*/*v*) agar and 1% (*w*/*v*) sucrose. For culturing NR-related mutant, the nitrogen in the MS medium included 1 mM NH_4_^+^ and 1.94 mM NO_3_^−^ [44]. Plates containing seeds were kept at 4 °C for 2 days, and then transferred into the growth chamber with a 16/8 h (23/21 °C) day/light regimes at 150 μmol·m^−2^·s^−1^ irradiation. Five-day-old wild-type (WT), and *noa1*, and *nia1/2* mutant plants grown on MS medium were treated with the indicated chemicals, as shown in legends. Three independent experiments with at least three replicates for each were performed, and about 80 samples were included in each replicate. Afterwards, Arabidopsis plants were photographed, and seedling leaf and root parts were measured immediately, or sampled for other analysis.

### 4.3. Determination of Melatonin by Enzyme-Linked Immunosorbent Assay (ELISA)

Melatonin was extracted from root tissues by using an acetone-methanol method, and determined by enzyme-linked immunosorbent assay (ELISA) [33,35]. After centrifugation at 12,000× *g* for 15 min at 4 °C, the extract was used for quantification of melatonin using the Melatonin ELISA Kit (Jiangsu Baolai Biotechnology, Nanjing, China).

### 4.4. Determination of NO by Griess Reagent

NO production in root tissues was determined by using Griess reagent [66,67]. Identical filtrate pretreated with 2-(4-carboxyphenyl)-4,4,5,5-tetramethylimidazoline-1-oxyl-3-oxide potassium salt (cPTIO), the specific scavenger of NO, for 15 min, was used as blanks. Absorbance was assayed at 540 nm, and NO content was calculated by comparison to a standard curve of NaNO_2_.

### 4.5. Laser Confocal Determination of Endogenous NO Production

Using a fairly specific NO fluorescent probe 4-amino-5-methylamino-2′,7′-difluorofluorescein diacetate (DAF-FM DA), the endogenous NO level of root tissues was detected by a TCS-SP2 confocal laser scanning microscopy (Leica Lasertechnik GmbH, Heidelberg, Germany; excitation at 488 nm, emission at 500–530 nm) [67,68]. Results are from five replicates per experiment. Fluorescence was expressed as relative fluorescence units using the Leica Confocal Software 2.5 (Leica Lasertechnik GmbH, Heidelberg, Germany).

### 4.6. ROS Detection

H_2_O_2_ and O_2_^−^ were histochemically detected by 3,3′-diaminobenzidine (DAB) and nitroblue tetrazolium (NBT) staining [35]. Finally, all samples were observed using a light microscope (model Stemi 2000-C; Carl Zeiss, Oberkochen, Germany).

### 4.7. Assay of Thiobarbituric Acid Reactive Substances (TBARS) Content

Oxidative damage was estimated by measuring the concentration of TBARS as previously described [69].

### 4.8. Determination of Antioxidant Enzyme Activities

Root tissues were crushed into fine powder in a mortar and pestle under liquid N_2_. Soluble proteins were extracted and homogenized in 50 mM PBS (pH 7.0) containing 1 mM EDTA and 1% polyvinylpyrrolidone (PVP), or with the addition of 1 mM ascorbic acid (ASA) in the case of ascorbate peroxidase (APX) activity determination. APX and SOD activities were measured as described previously [35,70]. Protein content was determined according to the method described by Bradford [71].

### 4.9. Real-Time Quantitative RT-PCR (qPCR) Analysis

According to our pervious method [35], total RNA was extracted from seedling roots using a Tranzol up kit (TransGen Biotech, Beijing, China). RNA concentration and quality were determined using the NanoDrop 2000 (Thermo Fisher Scientific, Wilmington, DE, USA), and then incubated with RNase-free DNase (TaKaRa Bio Inc., Dalian, China) to eliminate traces of DNA. cDNAs were then synthesized using an oligo(dT) primer and a SuperScript First-Strand Synthesis System (Invitrogen, Carlsbad, CA, USA).

By using the gene-specific primers (Supplementary Materials Tables S1 and S2), qPCR was conducted using a Mastercycler ep^®^ *realplex* real-time PCR system (Eppendorf, Hamburg, Germany) with *TransStart*^®^ Green qPCR SuperMix (TransGen Biotech, Beijing, China) [35]. In rapeseed plants, the expression levels of genes were normalized to two internal control gene *Actin* and *GADPH* transcript levels, and presented as values relative to corresponding control samples in NaCl-free conditions. In Arabidopsis, the expression levels of genes were normalized to two internal control gene *Actin* 2 and *GADPH* transcript levels, and presented as values relative to corresponding control of wild-type samples in NaCl-free conditions.

### 4.10. Determination of Ion Contents

Fresh seedling roots were harvested and washed four times by deionized water after treatments. According to the previous method [21,35], Na and K element contents were measured with an Inductively Coupled Plasma Optical Emission Spectrometer (ICP-OES, Perkin Elmer Optima 2100 DV; PerkinElmer, Shelton, CT, USA).

### 4.11. Quantification of Chlorophyll Content

Total chlorophyll was extracted using 95% (*v*/*v*) ethanol for 24 h in darkness, and then calculated by examining the absorbance at 649 nm and 665 nm [35].

### 4.12. Modified Biotin Switch Method

Modified biotin switch method was carried out according to the previous method [40,62]. After treatment, total protein was extracted from fresh seedling roots, and *S*-nitrosylated and biotin-labelled proteins were separated by using non-reducing sodium dodecyl sulfate polyacrylamide gel electrophoresis (SDS-PAGE; 12%). Western blotting was then performed to detect the proteins. Anti-biotin antibody horseradish peroxidase (Abcam antibodies, Cambridge, UK) was diluted 1:6000. Coomassie Brilliant Blue-stained gels were used to show that equal amounts of proteins were loaded.

### 4.13. Statistical Analysis

Values are means ± SE of three independent experiments with at least three replicates for each. Statistical analysis was performed using SPSS 16.0 software (IBM Corporation, Armonk, NY, USA). Differences among treatments were analyzed by one-way analysis of variance (ANOVA), taking *p* < 0.05 as significant according to Duncan’s multiple range test.

## Supplementary Materials

Supplementary materials can be found at http://www.mdpi.com/1422-0067/19/7/1912/s1. Supplementary data associated with this article can be found in the online version. Table S1: The sequences of primers for qPCR used in rapeseed. Table S2: The sequences of primers for qPCR used in Arabidopsis. Figure S1: NaCl-induced inhibition of root elongation was rescued by three types of NO-releasing compounds, but aggravated by the scavenger of NO.

## Abbreviations

ASA	Ascorbic acid
CAT	Catalase
cPTIO	2-(4-carboxyphenyl)-4,4,5,5-tetramethylimidazoline-1-oxyl-3-oxide potassium salt
DAB	3,3′-diaminobenzidine
GSNO	*S*-nitrosoglutathione
NBT	Nitroblue tetrazolium
*NHX1*	Sodium hydrogen exchanger
*noa1*	Nitric oxide associated1
NO	Nitric oxide
NONOate	Diethylamine
NR	Nitrate reductase
POD	Guaiacol peroxidase
PTIO	2-phenyl-4,4,5,5,-tetramethylimidazoline-1-oxyl-3-oxide
ROS	Reactive oxygen species
SNP	Sodium nitroprusside
SOD	Superoxide dismutase
*SOS*	Salt overly sensitive
