# Supplementary material for: Nitric Oxide Is Required for Melatonin-Enhanced Tolerance against Salinity Stress in Rapeseed (Brassica napus L.) Seedlings
Source: Int J Mol Sci. 2018 Jun 29;19(7):1912. doi: 10.3390/ijms19071912 (PMC6073977; doi:10.3390/ijms19071912)
Supplement: Supplementary file 1 [file ijms-19-01912-s001.pdf]

International Journal of Molecular Sciences

MDPI

Article

Nitric Oxide Is Required for Melatonin-Enhanced Tolerance against Salinity Stress in Rapeseed (*Brassica napus* L.) Seedlings

Gan Zhao [1], Yingying Zhao [1], Xiuli Yu [1], Felix Kiprotich [1], Han Han [1], Rongzhan Guan [2], Ren Wang [3] and Wenbiao Shen [1,*]

Supplementary Table 1 The sequences of primers for qPCR used in rapeseed.

Primer name	Accession number	Sequence (5′→3′)
Actin	AF111812	F: CTGACCGTATGAGCAAAG<br>R: CCACCGAACCAGAAGGCAGA
GAPDH	FJ529282	F: TTGGTGACAACAGGTCAAGCA<br>R: AAACTTGTCGCTCAATGCAATC
NHX1	AY189676	F: CAATGCAGGGTTTCAAGTA<br>R: AGTCAAAGGTCCCAATGTC
SOS2	AY310413	F: CCAGAGGTACTTAATGGCCAAG<br>R: GTGGCCTTGACTGAACAATGTA
APX	HE574697	F: GAGAAATACGCTGCTGATGAAGA<br>R: CCTACCCGAGCTCAGAAAGC
MnSOD	EF634058	F: TTGGTTCCTCTGGTGGGTATAGA<br>R: CAGATAATCCGGCCTCACATTC
Cu/ZnSOD	AY970822	F: CCAACGTCCACGCTGATAAGA<br>R: ACAGGTCCTATAGCCGTTGCA

Supplementary Table 2 The sequences of primers for qPCR used in Arabidopsis.

Primer name	Accession number	Sequence (5′→3′)
Actin 2	At3g18780	F: ACAACCGGTATTGTGCTGGA<br>R: GAAGACGGAGGATGGCATGA
GAPDH	At1g13440	F: TTGGTGACAACAGGTCAAGCA<br>R: AAACTTGTCGCTCAATGCAATC
SOS1	At2g01980	F: TGCTTGATGAGGGCAGAATA<br>R: ACTTGCGTGGGACAACTTTA
SOS2	At5g35410	F: TAGCGGAGAATGTAGAGAGAAAT<br>R: TGCTGTCGCCTGTCAAATAG
SOS3	At5g24270	F: TGATTGAAGTAATGGTGGATAAGGC<br>R: AGTCATGTTCTTGATGAGCGATG
APX1	At1g07890	F: CTCTGGGACGATGCCACAAG<br>R: CTCGACCAAAGGACGGAAAA
APX2	At3g09640	F: GTTCAGGATTCGAGGGTGC<br>R: GAAGAGCCTTGTCGGTTGGT
CAT	At1g20630	F: TTCCCTGTATTCTTCGTCCG<br>R: GCCTTCCATGTGCCTGTAGT
FSD1	At495100	F: AACCAGGTGGTGGAGGAA<br>R: GTAAGCAATGGGAAAGAGCC

International Journal of
Molecular Sciences

MDPI

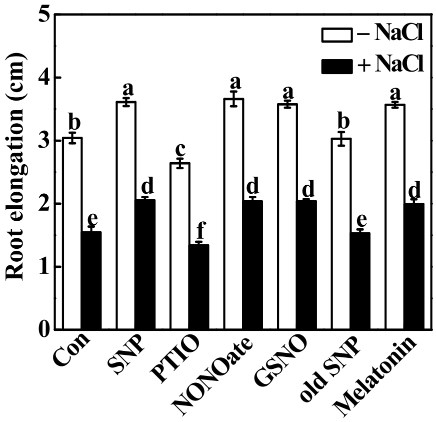

Figure S1. NaCl-induced inhibition of root elongation was rescued by three types of NO-releasing compounds, but aggravated by the scavenger of NO. Three-day-old rapeseed seedlings were pretreated with 10 μM SNP, 200 μM 2-phenyl-4,4,5,5-tetramethylimidazoline-1 -oxyl-3-oxide (PTIO; a scavenger of NO), 10 μM diethylamine NONOate (NONOate; a NO donor), 10 μM *S*-nitrosoglutathione (GSNO; a NO donor), 200 μM old SNP (a negative conrol of SNP), and 1 μM melatonin for 12 h, and then transferred to 200 mM NaCl for another 2 d. Afterwards, root elongation was measured. The sample without pretreatment was the control (Con). Values are mean ± SE of three independent experiments with at least three replicates for each. Bars with different letters are significant different at $p < 0.05$ according to Duncan's multiple range test.
